# Antichronic Gastric Ulcer Effect of Zinc-Baicalin Complex on the Acetic Acid-Induced Chronic Gastric Ulcer Rat Model

**DOI:** 10.1155/2018/1275486

**Published:** 2018-10-28

**Authors:** Hui Yang, Yi Lu, Xiao-Feng Zeng, Ling Li, Rong-Ping Zhang, Zhong-Kun Ren, Xu Liu

**Affiliations:** ^1^Biomedical Engineering Center, Kunming Medical University, Kunming 650500, China; ^2^Neurosurgery, The 1st Affiliated Hospital of Kunming Medical University, Kunming 650032, China; ^3^School of Forensic Medicine, Kunming Medical University, Kunming 650500, China

## Abstract

**Background:**

Baicalin (BA) has been shown to have anti-inflammatory and antioxidant activity. Zinc is a nutrient element.

**Objective:**

This study is aimed at investigating the antichronic gastric ulcer activity of Zn-Baicalin complex (BA-Zn) and its related mechanisms in an acetic acid-induced gastric ulcer rat model.

**Results:**

The severely ulcerated gastric mucosa of model rats had lower GSH-Px (52.21 ± 7.13) and SOD (7.03 ± 0.10) activity, and higher MDA (2.39 ± 0.03) content compared to sham rats. BA-Zn reduced the gastric ulcer index in a dose-dependent manner, significantly increased SOD activity and GSH-Px level, and reduced the MDA content and IL-8 and TNF-*α* levels in the gastric mucosa. BA-Zn (6.5 and 13 mg/kg) exerted a greater antiulcerogenic effect than both BA and zinc-gluconate, leading to a reduced ulcer index (18.43 ± 1.11, 15.00 ± 1.44), decreased MDA content (1.33 ± 0.07, 0.63 ± 0.01), and increased SOD activity (17.62 ± 0.11, 20.12 ± 0.32) and GSH-Px levels (102.12 ± 9.11, 120.25 ± 9.07). In addition, our results from Western blot suggested that BA-Zn (6.5 and 13 mg/kg) has a greater antiulcerogenic effect than both BA and zinc-gluconate.

**Conclusion:**

The BA-Zn complex possesses greater antichronic gastric ulcer properties compared to BA and zinc-gluconate due to its ability of oxidation resistance and anti-inflammatory effects.

## 1. Introduction

Peptic ulcers create a serious disease state in humans, are common worldwide, and have an increasing incidence rate. Gastric ulceration often increases the level of gastric acid secretion, can damage the gastric mucosal barrier, and penetrates the mucus layer [1, 2]. An ulcer model induced by acetic acid in rats resembles human chronic ulcers in both pathological features and healing process. Therefore, this model is more promising and useful for researchers to explore gastric ulcer. The pathogenesis of gastric ulcers is complex due to multifactorial and complex interaction between protective and destructive factors. Nonsteroidal anti-inflammatory drugs (NSAIDs) are the most commonly recommended medication and are comprehensively used to decrease clinical cases of pain and inflammation [3]. However, these drugs are known to induce intestinal ulcerations and hinder ulcer healing [4]. Traditional medicinal herbs and plants offer great promise in the treatment of many diseases and are an important source of new chemical substances with potential therapeutic effects to treat several diseases.

Chemical moieties isolated from plants have a long history of beneficial effects for treating ulcers. Baicalin (BA) is a flavonoid compound extracted and purified from the Chinese medicinal herb *Scutellaria baicalensis* Georgi, which has been demonstrated to have antiulcer, antipyretic, anti-inflammatory, analgesic, anticancer, antioxidant, and wound healing properties [5–10]. Zinc is essential for the maintenance of human cell functions. Moreover, it is reported that zinc can promote the healing of small intestinal mucosal damage. Zinc loss could significantly impair wound healing [11]. Zinc is also a free radical scavenger and anti-inflammatory agent, which can halt the progression of gastrointestinal disease and interrupt the associated inflammatory processes. Zinc complexes have always exerted potent antiulcer activity. Zinc-carnosine is a common antiulcer drug used in the treatment of gastric ulcers in Japan [12]. However, there is little research about the antichronic gastric ulcer ability of BA-Zn, and its related mechanism is still unclear.

It was reported that reactive oxygen species (ROS) played an important role in digestive disorders [13, 14]. In addition, oxygen free radical production and lipid peroxidation play an essential function in the development of gastric ulceration [15, 16]. Moreover, alterations to the antioxidant defense system in ulcerative disorders suggest a vital role for free radicals in ulcerations induced by acetic acid. Inflammatory cytokines such as IL-8 can also influence gastric ulcer healing [17, 18]. Thus, inflammatory cytokines are regarded as indicators of gastric ulcers.

In this study, we investigated whether the BA-Zn complex possesses antiulcer activity in a rat model of acetic acid-induced chronic gastric ulcer through free radical scavenging and anti-inflammatory activity.

## 2. Materials and Methods

### 2.1. Chemicals and Reagents

Baicalin (BA, Mw: 464.38) was purchased from Wanfang Chemical Company (Yunnan, China). The zinc complex of baicalin (BA-Zn, Zn(C_21_H_17_O_11_)(CH_3_COO)·3.5H_2_O, Mw: 632) was synthesized by the College of Pharmacy of Kunming Medical University. The LD50 of the zinc-baicalin (BA-Zn) complex is 4.76 g/kg·bw, and the ED50 is 1.042 mg/kg. IL-8, TNF-*α*, SOD, GSH-Px, and MDA kits were purchased from Nanjing Jiancheng Research Institute (Nanjing, China).

### 2.2. Preparation of BA-Zn

500 ml of water was added to 50 g of BA in a beaker, stirring constantly, and making this into a suspension liquid, heating to 55°C at pH 6.5–7.0, then adding a zinc acetate-saturated solution, and mixing fully. This was then cooled to room temperature, and filtered for red precipitation. This was then washed with 75% ethanol and ddH_2_O_2_, respectively, and finally drying these precipitation at 60°C.

### 2.3. Animals

Healthy male Sprague-Dawley rats, 7 weeks old and weighing 180–200 g, were from the experimental animal center of Kunming Medical College (certificate number SCXK2005-0008). The Animal Ethics Committee agreement number is KMMU2015011. The animals were housed on a pathogen-free environment with a constant 12 h light/12 h dark cycle in a temperature-controlled central facility (18–22°C) with free access to normal chow and tap water for a week. All procedures were in accordance with the Animal Ethics Committee of Kunming Medical University and the Guide for the Care and Use of Laboratory Animals.

### 2.4. Experimental Protocol for Ulceration and Healing

Animals were divided randomly into eight experimental groups with 10 animals in each group: the sham group (water vehicle, p.o. (*per os*)), model group (chronic gastric ulcers), BA-Zn groups (3.25, 6.5, and 13 mg/kg BA-Zn, p.o.), BA group (4.6 mg/kg BA, p.o.), zinc-gluconate group (9.32 mg/kg, p.o.), and omeprazole group (4.0 mg/kg omeprazole, p.o.). Chronic gastric ulcers were induced with acetic acid according to the method of Okabe et al. [19]. Briefly, the rats were anesthetized with sodium pentobarbital (30 mg/kg, respectively, i.p. (intraperitoneal injection)), and then the abdomen was opened, and the stomach exposed. A solution of 80% acetic acid (v/v, 0.5 ml) was instilled into a cylinder (5 mm of diameter) that was applied to the serosal surface of the stomach and, after 1 min, was removed by aspiration; the area of contact was washed with sterile saline. Forty-eight hours after ulcer induction, the rats were orally treated with vehicle (sham: water, 1 mg/kg), BA (4.6 mg/kg), zinc-gluconate (9.32 mg/kg), omeprazole (4.0 mg/kg), and BA-Zn (3.25, 6.5, 13 mg/kg) once a day for 7days. On the day following the last treatment, all animals were alive. Then, the animals were euthanatized by an overdose of pentobarbital sodium (150 mg/kg, i.p.), and the stomachs were removed and opened for the measurement of the ulcer area (mm^2^) as length (mm) × width (mm). The gastric ulcer tissue was used for the following tests.

### 2.5. Histological Preparation

A sample obtained from the middle of the gastric lesion was processed according to the conventional procedure and stained with hematoxylin-eosin. The stomachs were removed, filled with 5 ml of 10% formalin and allowed to stand for 5 min; then, these were cut open along the greater curvature. The longitudinal and abscissal lengths of the upper, opened part of the ulcer were measured with a micrometer that was attached to a stereoscopic microscope, and the product of both lengths (mm^2^) is expressed in terms of the ulcer index (Table 1). After the ulcer size was measured, the stomach tissue was again immersed in 10% formalin for 24 h. The formalin-fixed tissues were then cut so that a small amount of the normal tissue surrounding the ulcer remained.

### 2.6. Hematoxylin and Eosin Staining

The stomach tissues were immediately fixed in 4% paraformaldehyde for 48 h at 4°C. Paraffin sections (5 *μ*m) were prepared and then stained with hematoxylin and eosin (HE) according to standard procedures. Tissue were observed and microphotographed under a light microscope.

### 2.7. Detection of SOD Activity, GSH-Px, MDA, IL-8, and TNF-*α* Levels

Gastric tissue from the ulcerated portion of the stomach was excised, washed with distilled water, chopped, and homogenized at 3000 rpm in chilled Tris buffer (10 mM, pH 7.4) at a concentration of 10% w/v. Then, the homogenate was centrifuged at 9000 ×g at 4°C for 20 min and the supernatants were used for the determination of superoxide dismutase (SOD), methylenedioxyamphetamine (MDA), and GSH-Px (glutathione peroxidase) contents, as well as IL-8 and TNF-*α* expression levels by using the following detection kits purchased from Nanjing Jiancheng Biotechnology Co. Ltd.: a superoxide dismutase (SOD) assay kit (WST-1 method) (no. 20160914); glutathione peroxidase (GSH-Px) assay kit (colorimetric method) (no. 20160115); malondialdehyde (MDA) assay kit (TBA method) (no. 20161130); interleukin-8 assay kit (no. 20160815); and tumor necrosis factor-*α* assay kit (no. 20160703). The changes in absorbance were determined using a spectrophotometer; the results are expressed as U/l and *μ*mol/l for GSH-Px and MDA, U/mg protein for SOD activity, and ng/ml for IL-8 and TNF-*α* levels.

### 2.8. Western Blot Analysis

Western blot analysis was used for the protein expression levels of SOD1, TNF-*α*, and IL-8. Total proteins of tissues were extracted with lysis buffer (Beyotime, China). The insoluble protein lysate was removed by centrifugation at 10,000 ×g for 10 min at 4°C. Total proteins were separated by 10% sodium dodecyl sulfate-polyacrylamide gel electrophoresis (SDS-PAGE), followed by blocking in 5% skim milk on a PVDF membrane. The membranes were hybridized with primary antibodies (Proteintech, USA) for 2 h at 4°C and then with secondary antibodies (Proteintech, USA) for 2 h at room temperature. The bands were developed using an enhanced chemiluminescence (ECL) detection system (EMD Millipore, USA) for horseradish peroxidase (HRP). The relative amounts of the various proteins were analyzed. *β*-Actin was used as a loading control. The results were quantified using ImageJ software.

### 2.9. Statistical Analysis

All the results were expressed as mean ± SD. Statistical comparisons were made between drug-treated groups and acetic acid groups. The data was statistically analyzed by one-way analysis of variance (ANOVA) (Tukey's post hoc test) using GraphPad Prism software version 5.0a (GraphPad Software Inc., California, USA). *P* < 0.05 was considered to be statistically significant.

## 3. Results

### 3.1. BA-Zn Protects against Acetic Acid-Induced Gastric Lesions

**Figure 1:**
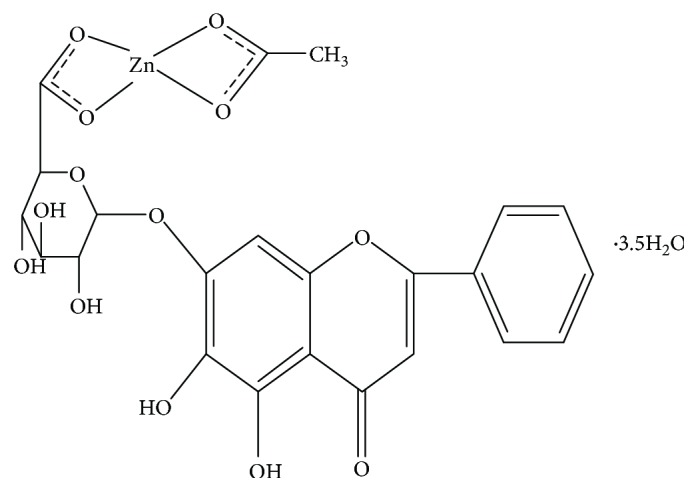
Structural formula of BA-Zn.

**Figure 2:**
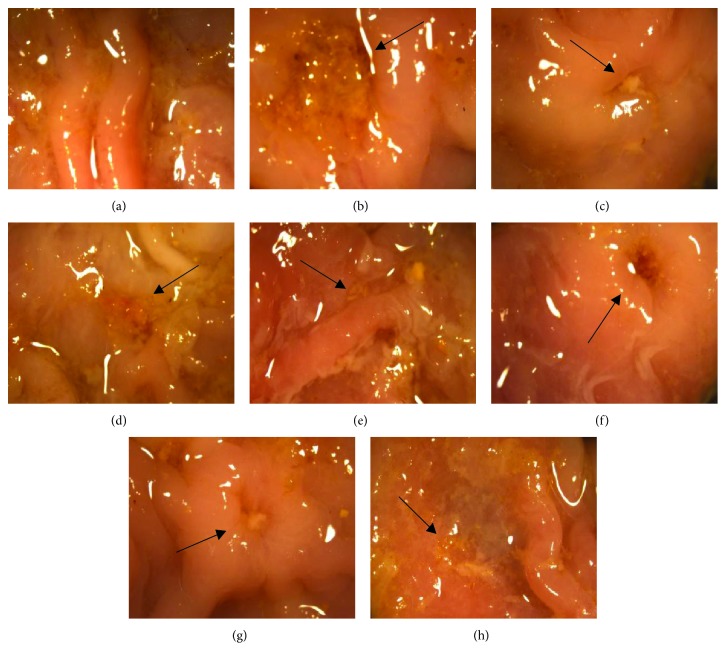
Appearances of acetic acid-induced gastric ulcers in different groups: (a) the sham group treated daily with water, (b) an ulceration from the model group induced by acetic acid, (c–e) an ulceration from the BA-Zn group induced by acetic acid and treated daily with BA-Zn (3.25, 6.5, and 13 mg/kg, p.o.), (f) an ulceration from the BA group induced by acetic acid and treated daily with BA (4.6 mg/kg, p.o.), (g) an ulceration from the zinc-gluconate group induced by acetic acid and treated daily with zinc-gluconate (9.32 mg/kg, p.o.), and (h) an ulceration from the omeprazole group induced by acetic acid and treated daily with omeprazole (4.0 mg/kg, p.o.). Black arrows indicate ulcer location.

The molecular formula of BA-Zn is Zn(C_21_H_17_O_11_)(CH_3_COO)·3.5H_2_O, and it has a molecular weight of 632 g/mol (Figure 1). The macroscopic appearance of the gastric tissue is shown in Figure 2. Rats treated with only water (Figure 2(a)) showed a normal glandular stomach.  Figure 2(b) shows the deep ulceration and severe edematous changes in the gastric mucosa of the model group that was induced with 80% acetic acid and treated with water daily. By contrast, rats treated with BA-Zn (3.25, 6.5, or 13 mg/kg) or BA, zinc-gluconate, or omeprazole showed less severe ulceration and less edema in the gastric mucosa induced by 80% acetic acid (Figures 2(c)–2(h)).

**Figure 3:**
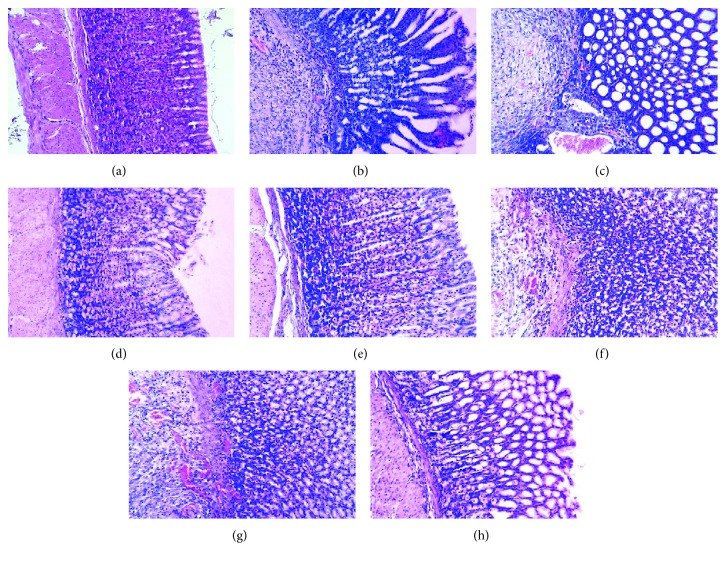
Photomicrographs of acetic acid-induced gastric ulcers from different groups: (a) sham group, (b) HE staining of the model group induced by acetic acid, (c–e) HE staining of the BA-Zn group induced by acetic acid and treated daily with BA-Zn (3.25, 6.5, and 13 mg/kg, p.o.), (f) HE staining of the BA group induced by acetic acid and treated daily with BA (4.6 mg/kg, p.o.), (g) HE staining of the zinc-gluconate group induced by acetic acid and treated daily with zinc-gluconate (9.32 mg/kg, p.o.), and (h) HE staining of the omeprazole group induced by acetic acid and treated daily with omeprazole (4.0 mg/kg, p.o.). The HE stained slides were visualized under a bright field microscope with 20x magnification.

As shown in Figure 3(a), the microscopic histological appearance of the gastric tissue and the normal gastric epithelium was observed in the sham group. The application of acetic acid induced the disruption of the superficial region of the gastric gland with loss of epithelial cells, as well as pronounced edema of the submucosa and degradation of the mucosa in the model group (Figure 3(b)).

BA-Zn (3.25, 6.5, and 13 mg/kg) and omeprazole markedly reduced the severity of gastric ulcers (swelling, destruction, and extensive destruction on the surface of the epithelium) and promoted marginal regeneration of the epithelium compared to the model group (Figures 3(c)–3(e) and 3(h)). In the BA (4.6 mg/kg) and zinc-gluconate- (9.32 mg/kg) treated groups, the severity of the gastric ulcers was also inhibited (Figures 3(f) and 3(g)).

In all animals from all the groups that survived, the longitudinal and abscissal lengths of the upper, opened part of the ulcer were measured with a micrometer that was attached to a stereoscopic microscope, and the product of both lengths (mm^2^) was expressed in terms of the ulcer index. As shown in Table 1, the sham group showed no ulcer index, and administration of BA-Zn at doses of 3.25, 6.5, and 13 mg/kg decreased the gastric ulcer index to 24.94 ± 2.00 (*P* < 0.05), 18.43 ± 1.11 (*P* < 0.001), and 15.00 ± 1.44 mm^2^ (*P* < 0.001) (12.0%, 35.0%, and 47.1% protection, respectively), compared to the ulcer index of the model group which was 28.35 ± 3.27 mm^2^. BA (4.6 mg/kg) reduced the gastric ulcer index to 24.38 ± 2.30 (*P* < 0.05) compared to that of the model group. Zinc-gluconate (9.32 mg/kg) also reduced the gastric ulcer index to 23.71 ± 2.00 (*P* < 0.05) compared to that of the model group. Moreover, zinc-gluconate had a higher gastric ulcer index than the BA-Zn group (6.5 and 13 mg/kg, *P* < 0.001, respectively). It indicated that BA-Zn (6.5 and 13 mg/kg) has a stronger healing effect than zinc-gluconate. Omeprazole (4.0 mg/kg), the positive control drug, demonstrated a potency for reducing the gastric ulcer index (15.35 ± 1.22 mm^2^; *P* < 0.001 compared to the model group). BA-Zn (13 mg/kg) showed the best potency for reducing the gastric ulcer index, which was the same as omeprazole.

### 3.2. BA-Zn Suppressed Oxidative Stress in Acetic Acid-Induced Gastric Tissue of Rats

Increased oxidative stress was shown in the ulcerated gastric mucosa of the model group, and this group also had significantly decreased SOD and GSH-Px activities and an increased level of lipid peroxidation (MDA) content compared to those of the sham group (*P* < 0.001).

As shown in Table 1, SOD activity in the mucosa of the model group was significantly lower (7.03 ± 0.10 U/mg protein) than in the sham group (22.09 ± 0.09 U/mg protein; *P* < 0.001). BA-Zn treatment at 6.5 and 13 mg/kg significantly increased the SOD activity to 17.62 ± 0.11 and 20.12 ± 0.32 U/mg protein (*P* < 0.001 and *P* < 0.001), respectively, compared to that of the model group (7.03 ± 0.10 U/mg protein). Moreover, the administration of 6.5 and 13 mg/kg BA-Zn led to higher SOD activity than did the administration of BA (17.62 ± 0.11 and 20.12 ± 0.32 vs. 12.09 ± 0.13 U/mg protein; *P* < 0.05 and *P* < 0.01), and these also markedly increased SOD activity than that of the zinc-gluconate group (9.07 ± 0.33 U/mg protein; *P* < 0.001). It indicated that BA-Zn has a stronger antioxidative effect than BA and zinc-gluconate. Zinc-gluconate (9.32 mg/kg) significantly increased SOD activity to 9.07 ± 0.33 U/mg protein (*P* < 0.05) compared to that of the model group (7.03 ± 0.10 U/mg protein). Omeprazole (4.0 mg/kg) demonstrated an increase in SOD activity (19.32 ± 0.09 U/mg protein; *P* < 0.001, compared to that of the model group).

GSH-Px levels in the gastric mucosa of the model group were significantly lower than in the sham group (52.21 ± 7.13 vs. 136.09 ± 9.09 U/l; *P* < 0.001). Administration of 6.5 and 13 mg/kg BA-Zn significantly increased the GSH-Px level in a dose-dependent manner (*P* < 0.001 and *P* < 0.001, respectively, compared to that of the model group). Moreover, the administration of 6.5 and 13 mg/kg BA-Zn led to increased GSH-Px activity compared to that of the BA group (102.12 ± 9.11 and 120.25 ± 9.07 vs. 85.07 ± 7.11 U/l; *P* < 0.05), and the administration of 6.5 and 13 mg/kg BA-Zn also markedly increased GSH-Px activity compared to that of the zinc-gluconate group (80.35 ± 6.77 U/l; *P* < 0.001). It indicated that BA-Zn has a stronger antioxidative effect than BA and zinc-gluconate. Zinc-gluconate (9.32 mg/kg) significantly increased GSH-Px activity to 80.35 ± 6.77 U/l (*P* < 0.05) compared to that of the model group (52.21 ± 7.13 U/l). Omeprazole (4.0 mg/kg) demonstrated an increase in GSH-Px activity (107.12 ± 9.05 U/l; *P* < 0.001 compared to that of the model group).

The inhibitory effects of BA-Zn on lipid peroxidation (MDA) are shown in Table 1. The MDA content in the gastric mucosa of the model group was higher than in the sham group (2.39 ± 0.03 vs. 0.91 ± 0.06 *μ*mol/l; *P* < 0.001). Treatment with 3.25, 6.5, and 13 mg/kg BA-Zn significantly decreased the MDA content to 1.75 ± 0.07, 1.33 ± 0.07, and 0.63 ± 0.01 *μ*mol/l (*P* < 0.01, *P* < 0.001, and *P* < 0.001, respectively, compared to that of the model group). Moreover, the administration of 3.25, 6.5, and 13 mg/kg BA-Zn led to lower MDA activity than BA (1.75 ± 0.07, 1.33 ± 0.07, and 0.63 ± 9.01 vs. 3.54 ± 0.04 *μ*mol/l; *P* < 0.05) and the zinc-gluconate group (2.00 ± 0.12 *μ*mol/l; *P* < 0.001), indicating that BA-Zn had a stronger antioxidative effect than BA and zinc-gluconate. Zinc-gluconate (9.32 mg/kg) showed a decrease in MDA content (2.00 ± 0.12 *μ*mol/l; *P* < 0.05 compared to that the model group). Omeprazole (4.0 mg/kg) also demonstrated a decrease in MDA content (1.00 ± 0.11 *μ*mol/l; *P* < 0.001 compared to that of the model group).

### 3.3. Effect of BA-Zn on the Concentration of TNF-*α* and IL-8

As shown in Table 2, the level of TNF-*α* in the model group was significantly higher than in the sham group (2.73 ± 0.10 vs. 0.72 ± 0.09 ng/ml; *P* < 0.001). The administration of 3.25, 6.5, and 13 mg/kg BA-Zn significantly decreased the TNF-*α* concentration in a dose-dependent manner (1.95 ± 0.06, 1.62 ± 0.11, and 1.12 ± 0.32; *P* < 0.05, *P* < 0.001, and *P* < 0.001, respectively, compared to that of the model group). Zinc-gluconate (9.32 mg/kg) demonstrated a decreased TNF-*α* level (2.00 ± 0.11 ng/ml; *P* < 0.05 compared to that of the model group). Administration of 6.5 and 13 mg/kg BA-Zn significantly decreased the TNF-*α* concentration compared to that of the zinc-gluconate group (2.00 ± 0.11 ng/ml; *P* < 0.001). Omeprazole (4.0 mg/kg) demonstrated a decreased TNF-*α* concentration (1.32 ± 0.09 ng/ml; *P* < 0.001 compared to that of the model group).

The level of IL-8 in the model group was significantly higher than in the sham group (0.69 ± 0.03 vs. 0.21 ± 0.06 ng/ml; *P* < 0.01). Administration of 3.25, 6.5, and 13 mg/kg BA-Zn significantly decreased the IL-8 level in a dose-dependent manner (0.36 ± 0.07, 0.33 ± 0.07, and 0.29 ± 0.01; *P* < 0.01, *P* < 0.01, and *P* < 0.001, respectively, compared to that of the model group). Zinc-gluconate (9.32 mg/kg) demonstrated a decreased IL-8 level (0.60 ± 0.01 ng/ml; *P* < 0.05 compared to that of the model group). Administration of 3.25, 6.5, and 13 mg/kg BA-Zn significantly decreased the IL-8 concentration compared to that of the zinc-gluconate group (0.60 ± 0.01 ng/ml; *P* < 0.001). Omeprazole (4.0 mg/kg) also demonstrated a decreased IL-8 level (0.45 ± 0.01 ng/ml; *P* < 0.05 compared to the model group).

**Figure 4:**
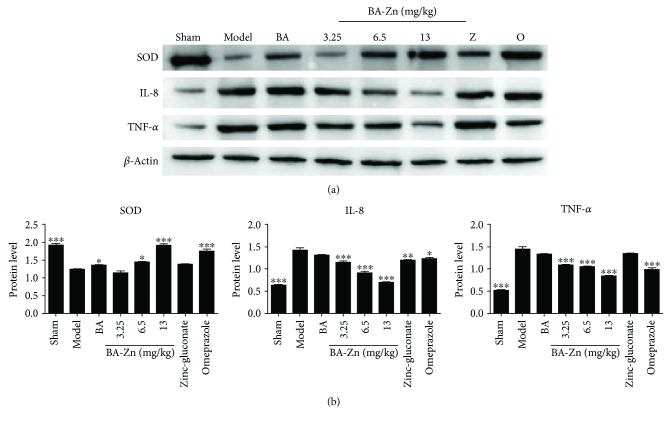
Western blot analysis of SOD, TNF-*α*, and IL-8 expression. (a) The protein expression levels of SOD, TNF-*α*, and IL-8 were analyzed by Western blot analysis. *β*-Actin was used as a loading control. (b) Quantification of SOD, TNF-*α*, and IL-8 protein expression levels using ImageJ software. Z: zinc-gluconate, O: omeprazole. ^∗^*P* < 0.05, ^∗∗^*P* < 0.01, and ^∗∗∗^*P* < 0.001, vs. the model group.

To further demonstrate the effect of BA-Zn on the concentration of SOD, TNF-*α*, and IL-8 in the above groups, Western blot assays were used to detect the expression levels of these proteins. In Figure 4(a) and 4(b), we reveal that BA-Zn treatment significantly increased SOD expression compared to that in the model and zinc-gluconate groups. BA-Zn also significantly decreased the expression of TNF-*α* and IL-8 compared to that in the model and zinc-gluconate groups. In summary, the above results indicate that BA-Zn had better antioxidative and anti-inflammatory effects than both BA and zinc-gluconate.

## 4. Discussion

Gastric ulcer is a common dysfunction of the digestive system, and an increasing number of people worldwide are suffering damage from ulcers. The acetic acid-induced ulcer model resembles human chronic ulcers in both pathological features and aspects of the healing process [20, 21]; thus, it is the most useful model for us to use when investigating gastric ulcers. Previous studies have demonstrated that experimental gastric ulcers have many histological and ultrastructural abnormalities, including a reduced height, marked dilation of gastric glands, and increased connective tissue, that may interfere with the mucosal defense system and cause ulcer recurrence with some ulcerogenic factors [22, 23].

In recent years, chemical drugs used for the treatment of gastric ulcers have induced many side effects. Recently, newly developed drugs with herbal origins may offer reduced side effects compared with chemical drugs [24]. In folk knowledge, medicinal plants have been used for the treatment of various disorders. More and more medicinal plants with various therapeutic properties, especially for the treatment of gastritis and gastric ulcers, have been identified [25–27]. However, few traditional Chinese medicinal plants for gastric ulcer treatment have been studied. Currently, because of the lack of effective and applicable pharmacological treatments for gastric ulcer, more and more people have focused on traditional medicines. The investigation of the therapeutic functions of traditional medicinal herbs in gastric ulcer offer promising potential.

*Scutellaria baicalensis* is a popular medicinal herb used in traditional Chinese medicine, and it is used for the treatment of high fevers, ulcers, inflammation, and even cancer. The main bioactive flavonoids in *Scutellaria baicalensis* include baicalein, baicalin (BA) (baicalein-7-glucuronide), wogonin, wogonoside (wogonin-7-glucuronide), oroxylin A, and oroxylin A-7-glucuronide. *Scutellaria baicalensis* and its flavones have been studied for their various pharmacological activities, including anti-inflammatory [28], antibacterial [29], neuroprotective [30], anticonvulsant [31], antiviral [32], antitumor [33], and antioxidant [34] activities. BA is the most popular bioactive flavonoid for treating ulcers. However, the antichronic gastric ulcer effects of BA alone or in complex have not yet been elucidated.

In this paper, we examined the effects of BA-Zn, BA, and zinc-gluconate and related mechanisms on gastric ulcer healing in an acetic acid-induced gastric ulcer rat model. It has been reported that zinc-gluconate has a protective effect on gastric ulcers [35]. Acetic acid induces a state of acute stress in the gastric mucosa, subsequently inducing gastric ulcers. Moreover, acetic acid-induced gastric ulceration leads to chronic oxidative stress with decreased SOD activity and GSH-Px expression levels and increased lipid peroxidation (MDA). It is well known that SOD, GSH-Px, and MDA play important roles in protecting the gastric mucosa against various damaging agents [36, 37]. GSH-Px also plays an important role in protecting against oxidative gastric mucosal injury [38]. MDA is currently regarded as a reliable index of ROS-induced mucosal injury [39]. Therefore, the detection of SOD, GSH-Px, and MDA contents can reflect and indicate the level of oxidative stress. In addition, increased concentrations of TNF-*α* and IL-8 were detected in the ulcerated gastric mucosa. BA-Zn (13 mg/kg) significantly accelerated the healing of gastric ulcers, through an increase in SOD and GSH-Px activity and a decrease in MDA, IL-8, and TNF-*α* contents. We also detected the levels of SOD, IL-8, and TNF-*α* by Western blot. We found that BA-Zn significantly accelerated ulcer healing by decreasing oxidative stress and attenuating inflammation. In our results, zinc-gluconate also has a protective effect on gastric ulcers, but BA-Zn has much stronger protective and healing abilities in gastric ulcers. On the basis of our data, BA-Zn (13 mg/kg) has greater antigastric ulcer abilities than either BA or zinc-gluconate.

Collectively, BA-Zn presents important ulcer-healing properties in the model of chronic ulcer induced by acetic acid in rats. Omeprazole is one of the most popular drugs used for the therapeutic control of gastroduodenal ulcers in humans. In our work, BA-Zn also exerted an antiulcerogenic effect similar to that of omeprazole against the development of acetic acid-induced gastric ulcers in rats using antioxidant and anti-inflammatory abilities. Therefore, the BA-Zn complex may become a potential and promising drug for the treatment of human gastroduodenal ulcers. In summary, our study indicates that BA-Zn promotes ulcer healing by decreasing oxidative stress and attenuating inflammation in an acetic acid-induced gastric ulcer rat model. However, additional studies are needed to explore the novel mechanisms of BA-Zn and determine whether other mechanisms are also involved in the antiulcer effects of this complex.

## 5. Conclusion

In conclusion, BA-Zn facilitates the healing of acetic-induced chronic ulcers in rats through its antioxidant and anti-inflammatory properties. The results of this study provide a foundation for the clinical application of BA-Zn in the treatment of gastroduodenal ulcers.

## Figures and Tables

**Table 1:** Effect of BA-Zn on oxidant stress in rat mucosa induced by acetic acid.

Group	(mg/kg)	Ulcer index (mm^2^)	SOD (U/mg)	MDA (*μ*mol/l)	GSH-Px (U/l)
Sham	—	0 ± 0^∗∗∗^	22.09 ± 0.09^∗∗∗^	0.91 ± 0.06^∗∗∗^	136.09 ± 9.09^∗∗∗^
Model	—	28.35 ± 3.27	7.03 ± 0.10	2.39 ± 0.03	52.21 ± 7.13
BA	4.6	24.38 ± 2.30^∗^	12.09 ± 0.13^∗∗^	3.54 ± 0.04^∗^	85.07 ± 7.11^∗∗^
BA-Zn	3.25	24.94 ± 2.00^∗^	6.75 ± 0.06	1.75 ± 0.07^∗∗^	55.11 ± 6.06
	6.5	18.43 ± 1.11^∗∗∗^	17.62 ± 0.11^∗∗∗^	1.33 ± 0.07^∗∗∗^	102.12 ± 9.11^∗∗∗^
	13	15.00 ± 1.44^∗∗∗^	20.12 ± 0.32^∗∗∗^	0.63 ± 0.01^∗∗∗^	120.25 ± 9.07^∗∗∗^
Zinc-gluconate	9.32	23.71 ± 2.00^∗^	9.07 ± 0.33^∗^	2.00 ± 0.12	80.35 ± 6.77^∗^
Omeprazole	4	15.35 ± 1.22^∗∗∗^	19.32 ± 0.09^∗∗∗^	1.00 ± 0.11^∗∗∗^	107.12 ± 9.05^∗∗∗^

^∗^ *P* < 0.05, ^∗∗^*P* < 0.01, and ^∗∗∗^*P* < 0.001, vs. the model group.

**Table 2:** Concentration of TNF-*α* and IL-8 in rat mucosa induced by acetic acid.

Group	(mg/kg)	TNF-*α* (ng/ml)	IL-8 (ng/ml)
Sham	—	0.72 ± 0.09^∗∗∗^	0.21 ± 0.06^∗∗^
Model	—	2.73 ± 0.10	0.69 ± 0.03
BA	4.6	1.91 ± 0.13^∗^	0.54 ± 0.04^∗^
BA-Zn	3.25	1.95 ± 0.06^∗^	0.36 ± 0.07^∗∗^
	6.5	1.62 ± 0.11^∗∗∗^	0.33 ± 0.07^∗∗^
	13	1.12 ± 0.32^∗∗∗^	0.29 ± 0.01^∗∗∗^
Zinc-gluconate	9.32	2.00 ± 0.11^∗^	0.60 ± 0.01^∗^
Omeprazole	4	1.32 ± 0.09^∗∗∗^	0.45 ± 0.01^∗^

^∗^ *P* < 0.05, ^∗∗^*P* < 0.01, and ^∗∗∗^*P* < 0.001, vs. the model group.

## Data Availability

The data used to support the findings of this study are available from the corresponding author upon request.
